# The ‘greater good’: Critical notes

**DOI:** 10.4103/0970-1591.33730

**Published:** 2007

**Authors:** An Ravelingien

**Affiliations:** Department of Philosophy, Bioethics Institute Ghent, Ghent University, Blandijnberg 2, 9000 Gent, Belgium. E-mail: an.ravelingien@ugent.be

Various regulatory authorities have gone to great lengths to find acceptable conditions under which xenotransplantation may proceed, rather than to preclude its development altogether. This is driven by the anticipation that if xenotransplantation will one day be successful, the overall benefits of this procedure will be immense. In principle, an unlimited supply of transplantable grafts could annul the current difficulties of ensuring equitable access to life-saving and/or ‘quality of life’-enhancing transplant activities. Proponents will feel that this potential benefit prevails over the unique ethical concerns related to the procedure - including animal welfare and public health issues. However, critical examinations of that anticipation are often left out of the analysis.

It is reasonable to say that the promise of xenotransplantation is not necessarily convincing. For instance, various arguments have arisen in the literature, which appear to undermine its appeal in terms of ‘saving more lives.’ A particular problem arises with the possibility that xenotransplantation will turn out to be no more than a temporary solution for patients with end-stage organ disease: a bridge to transplant. That is the primary utility of *ex vivo* perfusion techniques. The *in vivo* implantation of solid xenogeneic organs may also prove to be of limited duration, at least during the initial trial phases, if specific immunological rejection and physiological incompatibilities cannot be sufficiently overcome in advance. If xenotransplantation were merely to develop as a bridge to transplant, that would imply that the waiting lists for a human organ would not decrease, rather on the contrary. The same effect is expected for early use of totally implantable artificial hearts, as assessed by the Rathenau Institute.[1] A quantitative simulation model of the waiting list shows that if artificial hearts are introduced and provide only a short-term solution, more people on the waiting lists will die than would be the case if the normal donor heart program continued. This is because recipients of an artificial heart will, at some point, develop an acute need for an allotransplant. Given the urgency of the transplantation, those patients will be given priority on the waiting list, thereby directly lengthening others' time on the waiting list and indirectly affecting their mortality. Mortality will continue to increase unless the performance of the artificial heart almost equals that of a human heart. With a few exceptions (e.g., short-term liver perfusion may allow the liver to fully recover), it is reasonable to expect a similar increase in mortality until xenotransplantation becomes as successful as allotransplantation.

Before xenotransplantation achieves such qualitative standard, however, significant progress must still be made in countering the remaining stages of xenograft rejection and various pig-human physiological incompatibilities. Ringe *et al*. quote Thomas Starzl to support a justification of pursuing progress:

‘The future of xenotransplantation is brighter than at any previous time because what must be done to succeed has become remarkably clear.’[2]

While that may be the case, it is in no way clear that what must be done can be done. The optimism dates from the time in which there was unbound enthusiasm regarding the advances in the genetic manipulation of pigs to avoid hyperacute rejection. That enthusiasm led researchers to predict, as early as 1995, that clinical solid organ xenotransplants would be conducted within 5 years' time. Clearly, the feasibility of organ xenotransplantation has been seriously overestimated. Indeed, the many challenges that have hindered clinical success have made it very difficult for xenotransplant research programs to safeguard the high level of industry funding that was gained during the 1990s. By 2004, most biotech companies dedicated to overcoming hyperacute rejection by genetic modifications have effectively withdrawn from the field, reorganized their business alliance or greatly reduced their interest in xenotransplantation.[3]

As such, continued xenotransplantation research involves some exceptional costs. For instance, from the above it is clear that it has become increasingly dependent on federal funding. Furthermore, the type of research is distinctive in terms of the proportion of animals used and the level of suffering implied. Support for this alternative must not be led by uncritical expectations that it will save the day any time soon. Rather, it is of crucial importance that we do not lose sight of the responsibility to support alternative technologies or procedures that are equally dedicated to the ‘potential benefits’ of xenotransplantation but can nonetheless be pursued at a lesser overall cost.
